# Femoral artery injury during aneurysm coiling

**DOI:** 10.1590/0100-3984.2014.0081

**Published:** 2015

**Authors:** Daniel Aguiar-Dias, Luis Henrique Castro-Afonso, Daniel Giansante Abud

**Affiliations:** 1 Division of Interventional Neuroradiology, Department of Internal Medicine, Medical School of Ribeirão Preto, Universidade de São Paulo, Ribeirão Preto, SP, Brazil.

*Dear Editor*,

Endovascular artery reconstruction with low-profile stents, flow-diverters and flow-disrupting devices represent a significant progress in the endovascular therapy of intracranial aneurysms. Despite the improvement in technical expertise and developments in device technology, endovascular treatment still has inherent risks^(1)^. In the literature, most reports are focused on neurological complications during procedures^(2)^, however, reports on access vessel complications are scarce. Some of the well known access-related complications include: arterial pseudoaneurysms, arteriovenous fistulae, hematomas, arterial dissection leading to acute vessel occlusion^(3,4)^, intracavitary bleeding, and retroperitoneal hematoma following femoral artery puncture^(5)^. The authors report the case of a large groin hematoma caused by a hypodermic needle connected with the black cable of the detachable coil power supply (Boston Scientific; Natick, MA, USA) and its endovascular management.

Local compression is the first line treatment for femoral access complications^(6)^, but such strategy may fail when indicated for patients under combined antiplatelet and anticoagulation regimens. Open surgery is effective in the treatment of groin complications^(7)^. However, the endovascular approach is a safe and effective minimally invasive alternative to surgery in the management of access vessel complications^(8-10)^. A bleeding originated from an arterial access can be treated by endovascular approach using either liquid or coil embolization^(8,9)^, or by stent-graft implantation^(10)^. In the present case, selective embolization was performed with N-butyl-2-cyanoacrylate because the bleeding site was located in a thin distal branch of the right deep femoral artery, allowing for micro-catheterization and injection of a liquid embolic agent. In the present case, the very small caliber of the bleeding vessel precluded the use of coils. Moreover, because the bleeding was located in a distal branch of the deep femoral artery, and not in the wall of the artery, there was the option for selective embolization instead of stent-graft placement. Selective arterial embolization avoids potential risks associated with a stent-graft implantation, such as thrombosis, kinking, compression, occlusion of femoral branches and long-term stent occlusion.

**Figure 1:**
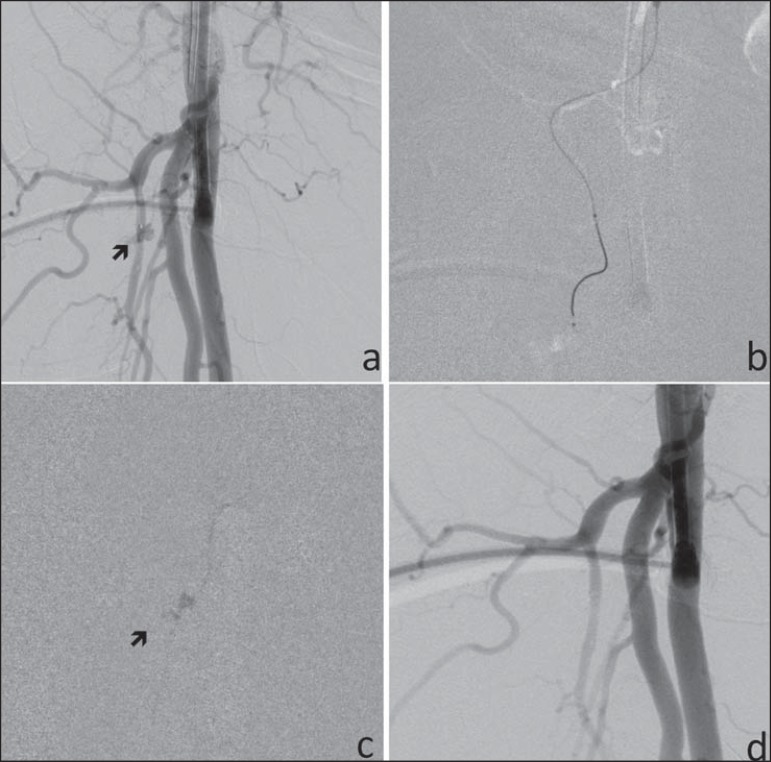
**a:** Angiography of right femoral artery (RFA) shows the 7F sheath inserted into the common femoral artery and an active contrast extravasation (arrow) in a small branch of the deep RFA, consistent with active bleeding. **b:** Microcatheter placed into a small muscular branch of the deep RFA. **c:** Microcatheter for glue injection into the small muscular branch of the deep RFA and contrast extravasation (arrow). d: Post-treatment RFA angiography shows absence of bleeding.

The present case reveals an unexpected complication during aneurysm embolization and alerts us to the possibility of uncommon bleedings secondary to simultaneous use of aspirin, clopidogrel, and unfractioned heparin.
